# Cost-Effectiveness Analysis of Different Testing Strategies that Use Antibody Levels to Detect Chronic Hepatitis C in Blood Donors

**DOI:** 10.1371/journal.pone.0154625

**Published:** 2016-05-09

**Authors:** Víctor Granados-García, Ana M. Contreras, Carmen García-Peña, Guillermo Salinas-Escudero, Hla-Hla Thein, Yvonne N. Flores

**Affiliations:** 1 Unidad de Investigación Epidemiológica y en Servicios de Salud Área Envejecimiento, Instituto Mexicano del Seguro Social, Ciudad de México, México; 2 Departamento de Salud Pública. Centro Universitario de Ciencias de la Salud, Universidad de Guadalajara, Guadalajara Jalisco, México; 3 Dirección de Investigación, Instituto Nacional de Geriatría, Ciudad de México, México; 4 Centro de Estudios Económicos y Sociales en Salud, Hospital Infantil de México Federico Gómez, Ciudad de México, México; 5 Dalla Lana School of Public Health, University of Toronto, Toronto, Canada; 6 Unidad de Investigación Epidemiológica y en Servicios de Salud, Instituto Mexicano del Seguro Social, Cuernavaca Morelos, México; 7 UCLA Department of Health Policy and Management, Fielding School of Public Health and Jonsson Comprehensive Cancer Center, Los Angeles, CA, United States of America; Università degli Studi di Palermo, ITALY

## Abstract

Aim. We conducted a cost-effectiveness analysis of seven hepatitis C virus (HCV) testing strategies in blood donors. Methods. Three of the seven strategies were based on HCV diagnosis and reporting guidelines in Mexico and four were from previous and current recommendations outlined by the CDC. The strategies that were evaluated determine antibody levels according to the signal-to-cut-off (S/CO) ratio and use reflex Immunoblot (IMB) or HCV RNA tests to confirm true positive (TP) cases of chronic HCV infection. Costs were calculated from the perspective of the Mexican Institute of Social Security (IMSS). A decision tree model was developed to estimate the expected number of true positive cases and costs for the base-case scenarios and for the sensitivity analyses. Results. Base-case findings indicate an extended dominance of the CDC-USA2 and CDC-USA4 options by the IMSS Mexico3 and IMSS-Mexico1 alternatives. The probabilistic sensitivity analyses results suggest that for a willingness-to-pay (WTP) range of $0–9,000 USD the IMSS-Mexico1 strategy is the most cost-effective of all strategies ($5,000 USD per TP). The IMSS-Mexico3, IMSS-Mexico2, and CDC-USA3 strategies are also cost-effective strategies that cost between $7,800 and $8,800 USD per TP case detected. The CDC-USA1 strategy was very expensive and not cost-effective. Conclusions. HCV antibody testing strategies based on the classification of two or three levels of the S/CO are cost-effective procedures to identify patients who require reflex IMB or HCV RNA testing to confirm chronic HCV infection.

## Introduction

Hepatitis C virus (HCV) infection is a serious public health problem [1]. Approximately one-third of chronically infected HCV patients will develop cirrhosis or hepatocellular carcinoma, but most will remain asymptomatic until complications arise [2, 3]. Globally, there has been a dramatic improvement in HCV testing over the past decade with the development of a variety of serologic assays that detect HCV antibodies and molecular assays that detect or quantify HCV ribonucleic acid (HCV RNA) [4]. An HCV infection diagnosis begins with the detection of hepatitis C antibody (Anti-HCV) levels, and a reactive or positive antibody result is followed up with supplemental testing to confirm the presence of an infection [5, 6]. Strategies to detect HCV primarily serve as a means to confirm or rule out chronic infection based on a positive Anti-HCV result. In clinical practice, the choice of which supplemental test to use, such as Immunoblot (IMB) or HCV RNA, depends on several factors such as the patient’s symptoms, the clinician’s preference, the availability of supplemental testing in public and private laboratories, and the cost of the tests [7].

In 2003, the Centers for Disease Control and Prevention (CDC) published recommendations for laboratory testing and the reporting of Anti-HCV results, with the option to classify the positive antibodies as low or high, and then choose supplementary testing based on the signal-to-cut-off (S/CO) ratio [5]. The CDC guide also proposed that cases which could be classified as having true positive results with a high level of Anti- HCV should be confirmed by IMB[5]. Our group developed a guideline as a recommendation for IMSS (Mexico) that classifies and reports positive HCV antibodies according to three levels (very low, low, and high), depending on the cut-off point[7, 8]. The authors propose that the use of a high antibody level classification is a practical and accurate serological marker for detecting viremia. This guideline recommends routine HCV RNA testing for those with a high Anti-HCV result in order to confirm a chronic HCV infection [7]. Antibodies are detected by immunoassays and the antibody level is determined semi-quantitatively by the cut-off ratio and must be interpreted “numerically”. Samples with S/CO ratio <1 are classified as negative and samples with S/CO ratio ≥1 as reactive or positive.

Recently, the CDC guidelines were updated to include the recommendation that all persons with a positive Anti-HCV result should be evaluated for the presence of HCV RNA in their blood, regardless of the antibody level [6]. Among blood donors or the general population, viremia is only detected in 30 to 40% of subjects with a positive antibody result [5, 8, 9]. In that situation, a high antibody level can differentiate patients who are viremic from those who are not, regardless of population characteristics or prevalence of HCV. The decision to use a specific supplemental testing strategy has economic implications for society. In health care settings with limited budgets, the recommendation to always use HCV RNA testing might not be feasible and could result in a waste of limited resources in countries with a low prevalence of HCV infection (less than 2%).

A review of the literature was conducted using MEDLINE, EMBASE, and Science Citation Index Expanded to identify the latest economic analyses on strategies to diagnose HCV in positive antibody patients using the following search terms: costs, hepatitis C, hepatitis C virus, and diagnosis. We identified five economic studies of diagnosis alternatives [10–14], and all except one were conducted in high-income countries.

Chapko et al. evaluated a set of strategies to detect chronic HCV infection using a decision analysis. The strategies evaluated by these authors use an immunoassay with antibody results classified in 1–3 levels followed by IMB or HCV RNA [12]. The main finding was that the strategy using IMB is preferable when the prevalence of HCV infection is below 20% [12]. According to the authors, when prevalence increases, opting to use IMB and HCV RNA instead of only HCV RNA will depend on the relative importance of avoiding one false positive vs. missing one true positive. While we consider these results to be relevant, the cost per true positive (TP) is a more direct result that may be easier to interpret in economic terms than the trade-off measure estimated by Chapko. Therefore, the objective of this research was to conduct a cost effectiveness analysis of seven strategies for diagnosing chronic HCV in a low prevalence Mexican population, using the aforementioned cost per TP.

## Material and Methods

A cost-effectiveness analysis was undertaken to evaluate seven strategies for the diagnosis of chronic HCV infection in a low prevalence population [9]. This evaluation was conducted from the perspective of the health care provider (payer) and considers a time horizon of one year. The outcome of the analysis was the proportion of true positive (TP) cases detected. TP cases were defined using the following three clinical conditions that were established based on the results of the three tests used in the diagnostic procedure: (1) a high Anti-HCV test defined by the S/CO≥20 and a positive HCV RNA test; (2) the results of both the IMB (RIBA) and HCV RNA tests were positive; and (3) a positive IMB test although the HCV RNA test was negative. The third option (when the IMB rest is positive but the RNA HCV test is not), generally occurs in a limited number of cases. However, this is a relevant outcome because the IMB test cannot distinguish between a current infection (viremia) and a past infection, in which the individual could still be a potential virus carrier even if the infection was resolved (7).

We evaluated seven diagnosis strategies, four that are based on CDC recommendations [5, 6] and three strategies that are recommended for the IMSS [7] to identify viremia in persons with a positive antibody test. The classification levels for a positive or reactive Anti-HCV result were established according to previously published papers based on the signal to cut off (S/CO) ratio [5, 6]. Each alternative consists of a combination of the initial Anti-HCV test and one or two of the supplementary tests: IMB and/or HCV RNA (Table 1).

**Table 1:** Description of strategies for cost-effectiveness analysis based on the anti-HCV level classification.

Strategy	Anti-HCV level †	Supplementary tests	Testing sequence
CDC-USA1	≥1	HCV RNA and IMB	HCV RNA→ IMB
CDC-USA2	≥1	HCV RNA and IMB	IMB→HCV RNA
CDC-USA3	High (≥8)	HCV RNA and IMB	HCV RNA→ IMB
CDC-USA3	Low (≤8)	HCV RNA and IMB	IMB→HCV RNA
CDC-USA4	≥1	Only HCV RNA	HCV RNA
IMSS-Mexico1	High (≥20)	HCV RNA and IMB	HCV RNA→ IMB
IMSS-Mexico1	Low (4.5≤S/CO<20)	HCV RNA and IMB	IMB→HCV RNA
IMSS-Mexico1	Very low (1≤ S/CO<4.5)	No	No testing
IMSS-Mexico2	High (≥20)	HCV RNA and IMB	HCV RNA→ IMB
IMSS-Mexico2	Low (4.5≤S/CO<20)	HCV RNA and IMB	IMB→HCV RNA
IMSS-Mexico2	Very low (1≤ S/CO<4.5)	HCV RNA and IMB	IMB→HCV RNA
IMSS-Mexico3	Low (<20)	HCV RNA and IMB	IMB→HCV RNA
IMSS-Mexico3	High (≥20)	HCV RNA and IMB	HCV RNA→ IMB

Abbreviations: Anti-HCV: Antibody tests for HCV, IMB: Immunoblot test, HCV RNA: RNA tests for HCV.

^†^ The Anti-HCV level is based on the Signal-to-cut-off (S/CO) ratio of antibodies concentration

**Fig 1:**
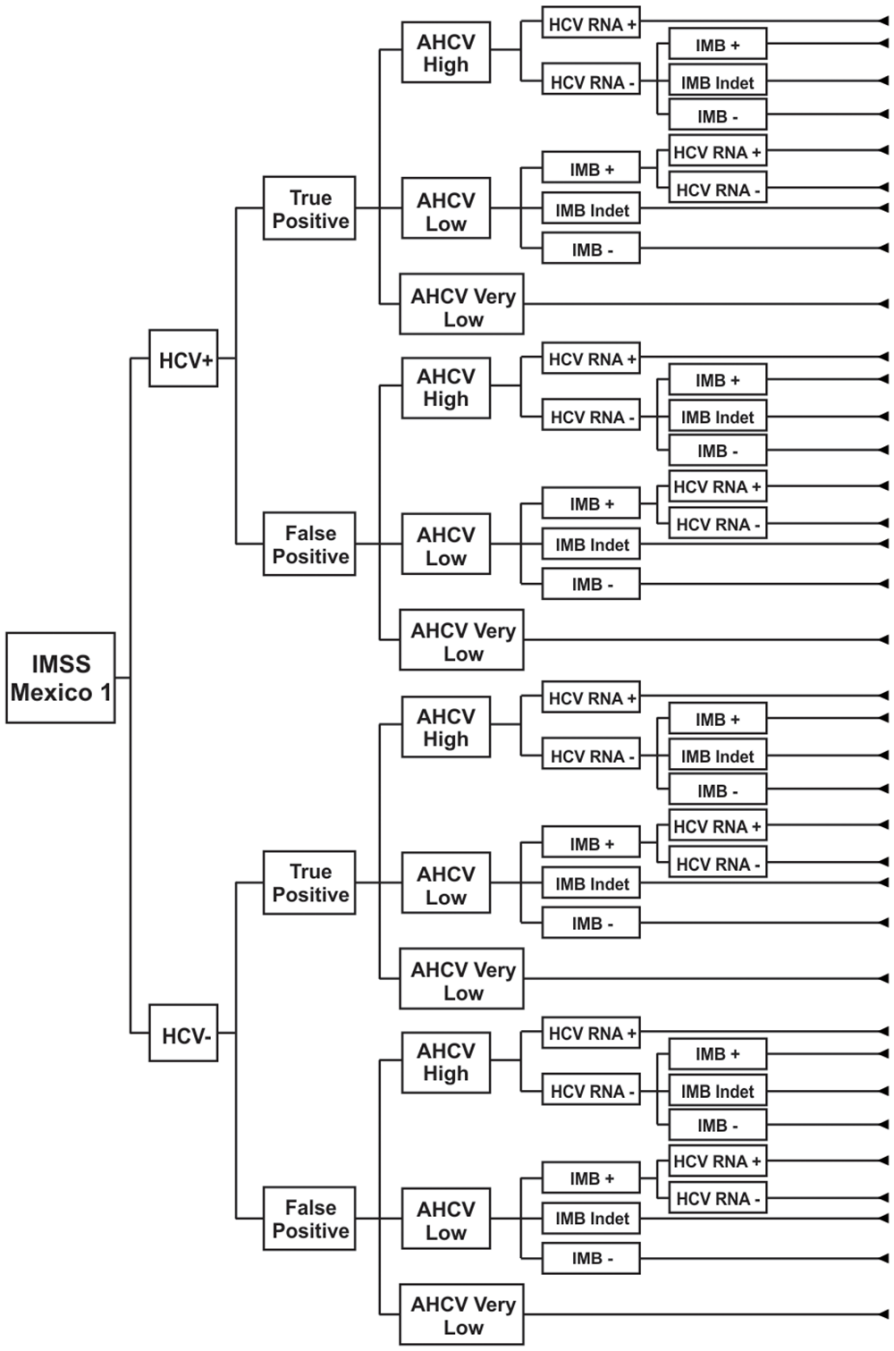
Decision tree for the IMSS-Mexico1 strategy. The decision tree describes first the situation of being HCV+ or HCV-, then situation of being a TP or a FP, then estimates the expected value of for each strategy of the tree.

A decision tree model was created to estimate the expected number of TP cases and the associated costs of each diagnosis strategy (Fig 1) [9]. We used a decision tree model that is similar to the structure reported by Chapko et al. [12], but we used the proportion of TP cases as an outcome (instead of HCV positive or HCV negative, viremic or not viremic cases), since our objective was to estimate the cost per TP case detected and not the global sensitivity and specificity of the negative and positive cases.

In its report of the modeling good research practice task force, the International Society for Pharmacoeconomics and Outcomes Research and the Society for Medical Decision Making (ISPOR-SMDM) classify models as either multipurpose in function or as more specific to a single purpose. This report also provides approaches for evaluating variability in models and provides recommendations for transparency in the reporting of a model [15]. The specific purpose of our model is to evaluate a combination of diagnostic tests. In order to be transparent in the reporting of our model, first we provided a non-technical description that explains what our model is and the results it generated, and second, we documented how we constructed the model and the values of the parameters we used, as well as our assumptions.

We assumed an HCV prevalence of 0.014 [16] and a proportion of TP cases of 0.3759 [9] as fixed parameters in the model for all the diagnostic strategies. The probability of the HCV RNA test being positive when a patient is viremic (sensitivity) and the probability of the test being negative when the patient is not viremic (specificity) were both assumed to be one. The other probabilities used in the model were estimated from the results of a study among blood donors that was conducted by Contreras et al. [9]. The procedure to determine the probabilities was based on the aforementioned definitions of TP and FP cases and the established level of the S/CO index, which were used to create a decision tree that considers the sequence of the diagnostic tests. For example, if the total number of TP cases was 244 and this amount included all three levels of S/CO (S/CO≥20 = 208; 4.5≤S/CO<20 = 29 and 1≤S/CO<4.5 = 7), the probability of having an S/CO≥20 result would be estimated as 208/244 = 0.8525. The sensitivity and specificity of the HCV RNA test was assumed to be 100%, which we varied between 98–100% to observe the effect of a small variation for the deterministic and probabilistic sensitivity analyses (Table 2). We assumed that the conditional probabilities are independent and mutually exclusive.

**Table 2:** Parameters values and intervals for sensitivity analysis.

Parameters	Base-case value^†^	Sensitivity analysis interval
Probability HCV-RNA+ when viremic	1.0000	0.98–1.00
Probability HCV-RNA+ when not viremic	0.0001	0.0–0.0002
Probability S/CO ≥ 20 when TP	0.8525	0.845–0.859
Probability 4.5≤S/CO<20 when TP	0.1189	0.112–0.125
Probability S/CO ≥ 8 when TP	0.8754	0.855–0.895
Probability IMB+ when TP and S/CO ≥ 20	0.9904	0.984–0.996
Probability IMBIndet when TP and S/CO ≥ 20	0.0096	0.008–0.012
Probability IMB+ when TP and 4.5≤S/CO<20	1.0000	0.98–1.00
Probability IMBIndet when TP and 4.5≤S/CO<20	0.0000	0.0–0.0001
Probability IMB+ when TP and S/CO 1≤S/CO<4.5	1.0000	0.98–1.00
Probability IMBIndet when TP and 1≤ S/CO<4.5	0.0000	0.0–0.0001
Probability IMB+ when TP and S/CO <20	1.0000	0.98–1.00
Probability IMBIndet when TP and S/CO <20	0.0000	0.0–0.0001
Probability IMB+ when TP and S/CO ≥1	0.9918	0.985–0.997
Probability IMBIndet when TP and S/CO ≥1	0.0082	0.003–0.014
Probability IMB+ when TP and S/CO ≥8	0.9914	0.990–0.993
Probability IMBIndet when TP and S/CO ≥8	0.0086	0.003–0.014
Probability IMB+ when TP and S/CO <8	1.0000	0.98–1.00
Probability IMBIndet when TP and S/CO <8	0.0000	0.0–0.0001
Probability S/CO ≥ 20 when FP	0.0101	0.008–0.012
Probability S/CO 4.5≤S/CO<20 when FP	0.1924	0.185–0.200
Probability S/CO ≥ 8 when FP	0.0835	0.078–0.089
Probability IMB+ when FP and S/CO ≥ 20	0.0000	0.0–0.0001
Probability IMB Probability IMBIndet when FP and S/CO ≥ 20	0.5000	0.49–0.51
Probability IMB+ when FP and 4.5≤S/CO<20	0.0000	0.0–0.0001
Probability IMBIndet when FP and 4.5≤S/CO<20	0.4078	0.398–0.417
Probability IMB+ when FP and 1≤ S/CO<4.5	0.0000	0.0–0.001
Probability IMBIndet when FP and 1≤ S/CO<4.5	0.2825	0.274–0.291
Probability IMB+ when FP and S/CO <20	0.0000	0.0–0.0001
Probability IMBIndet when FP and S/CO <20	0.3069	0.298–0.316
Probability IMB+ when FP and S/CO >1	0.0000	0.0–0.0001
Probability IMBIndet when FP and S/CO >1	0.3088	0.300–0.318
Probability IMB+ when FP and S/CO ≥8	0.0000	0.0–0.0001
Probability IMBIndet when FP and S/CO ≥8	0.3939	0.384–0.403
Probability IMB+ when FP and S/CO <8	0.0000	0.0–0.0001
Probability IMBIndet when FP and S/CO <8	0.3011	0.292–0.310
Cost Anti-VHC †	10.44	-+20%
Cost Immunoblot (IMB)	126.27	-+20%
Cost VHC RNA	232.01	-+20%

Abbreviation meanings TP: True positive; FP: false positive; IMB: Immunoblot; IMBIndet: Indeterminate result of IMB test. Information of costs is in 2014USD.

^†^ Sources of information are as follows: The probability of the HCV RNA test being positive when viremic (sensitivity) and being negative when not viremic (specificity) was assumed to be one. Other probabilities were estimated based on a previous study reporting data on results in 640 blood donors [9](9). Costs of tests were taken from [7](7).

The costs of the seven diagnostic alternatives that were evaluated included the direct costs associated with Anti-HCV, IMB and HCV RNA tests. (Table 2) The cost of each test was valued at the institutional price paid by IMSS to its suppliers based on payment-per-testing data that was previously reported (7). The total cost of each test includes the necessary supplies and reagents, as well as the computer equipment, software and all required materials to obtain, register, and report the results. (Table 2) All costs were converted and reported to prices of the year 2014 then converted to US dollars (USD) [17, 18].

Deterministic and probabilistic sensitivity analyses (DSA and PSA) were conducted to evaluate the effect of varying the parameters to represent uncertainty in the model and to examine the robustness of numerical results with respect to input parameters. For these analyses, we estimated a confidence interval level of 95% and used the formula for proportions [19]. A one-way sensitivity analysis that included all probabilities is reported as a tornado diagram.

To conduct the PSA we assumed a gamma inverse distribution for the costs and a normal distribution for the conditional probabilities. A Monte Carlo simulation with 1,000 iterations of the joint distribution of all parameters was conducted to map the uncertainty across the cost and effectiveness parameters. The results of this simulation are presented as cost-effectiveness acceptability curves (CEACs).

## Results

**Fig 2:**
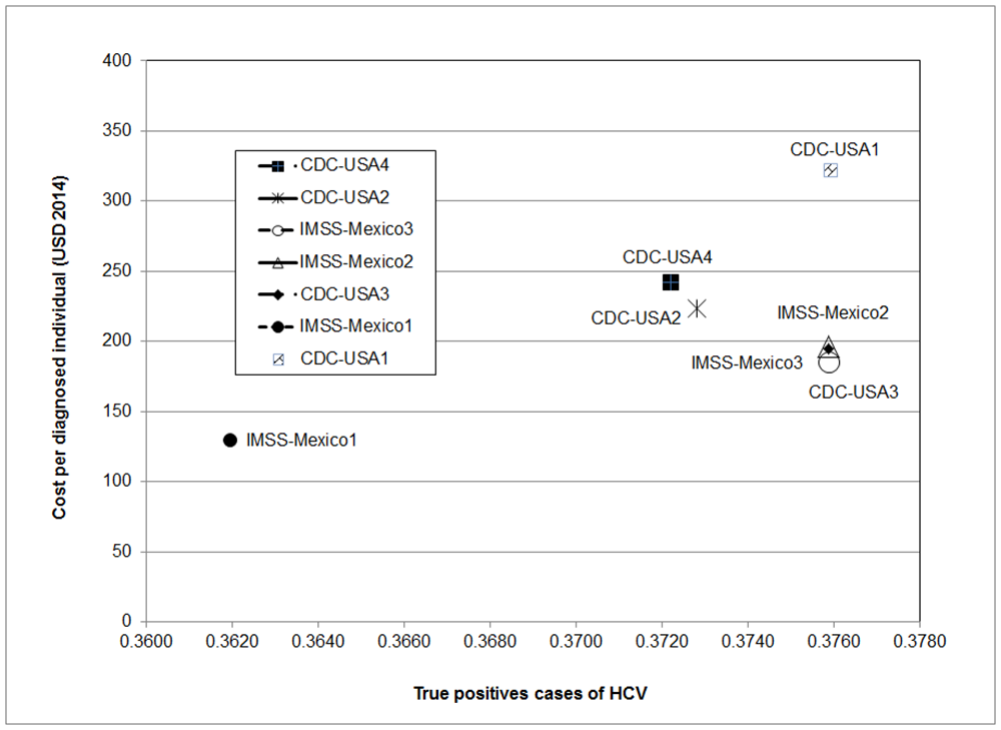
Base-case expected costs and portion of TP cases for diagnostic strategies. These are the results in base case estimates. Strategies IMSS-Mexico1, IMSS-Mexico2 and CDC-USA3 have almost the same effectiveness and costs. IMSS-Mexico1, IMSS-Mexico3 and CDC-USA1 are in the efficiency frontier.

The IMSS-Mexico1 strategy has the lowest cost per individual diagnosed ($130 USD) and is the least effective (0.3619) (Table 3). The next most effective option is the CDC-USA4 strategy (0.3722) but it is more costly ($242 USD) per TP case detected. When compared with the other strategies, the CDC-USA2 and CDC-USA4 alternatives are dominated by the IMSS-Mexico1 and IMSS-Mexico3 options (Extended dominance) (Fig 2).

**Table 3:** Costs, health results and cost-effectiveness estimates of strategies in base-case scenario.

Strategy	Cost †	HR ‡	I-Costs §	I-HR §	ACER †	IICER †	Dominance
IMSS-Mexico1	$130	0.361955			$359		Undominated
CDC-USA4	$242	0.372203	$113	0.010248	$651	$11,027	Dominated ^a^
CDC-USA2	$223	0.372818	$-19	0.000615	$599	Negative	Dominated ^a^
IMSS-Mexico3	$185	0.375869	$-38	0.003051	$493	Negative	Undominated
IMSS-Mexico2	$197	0.375875	$11	0.000006	$523	$1,833,333	Dominated ^a^
CDC-USA3	$195	0.375877	$-2	0.000002	$518	Negative	Undominated
CDC-USA1	$322	0.375932	$127	0.000055	$856	$2,309,091	Undominated

^†^ Costs are in 2014USD.

^‡^ Health result (HR) was measured in TP cases defined as follows: 1) High level Anti-HCV test with HCV RNA positive test, 2) IMB positive and HCV RNA positive and 3) IMB Indeterminate and HCV RNA positive.

^§^ Incremental Costs (I-Costs) and Incremental HR (I-HR) were estimated considering the immediate less effective alternative.

¶ Undominated strategy means that the previous alternative is less effective and costs less (IMSS-Mexico3 vs IMSS-Mexico1). A dominated strategy is when the previous strategy costs less costs and is more effective (IMSS-Mexico3 vs CDC-USA2).

^a^ Extended dominance is when there are two alternatives which are less costly and more effective than the considered strategy (IMSS-Mexico1 and IMSS-Mexico3 vs CDC-USA2).

The ICER of the IMSS-Mexico3 strategy compared with the IMSS-Mexico1 is part of the efficiency frontier (Fig 2). Here we can see that CDC-USA2 and CDC-USA4 strategies are dominated. The IMSS-Mexico2 strategy appears to be slightly more cost effective than the IMSS-Mexico3 option, although the small difference in effectiveness (6x10^-6^) appears to not be significant. The CDC-USA3 strategy is also similar to the IMSS-Mexico3 alternative, with a cost difference of only $10 USD and an insignificant difference in effectiveness (8x10^-6^). In the base case scenario, the CDC-USA1 strategy appears to be significantly more expensive and more effective than CDC-USA3, but with a much greater ICER.

**Fig 3:**
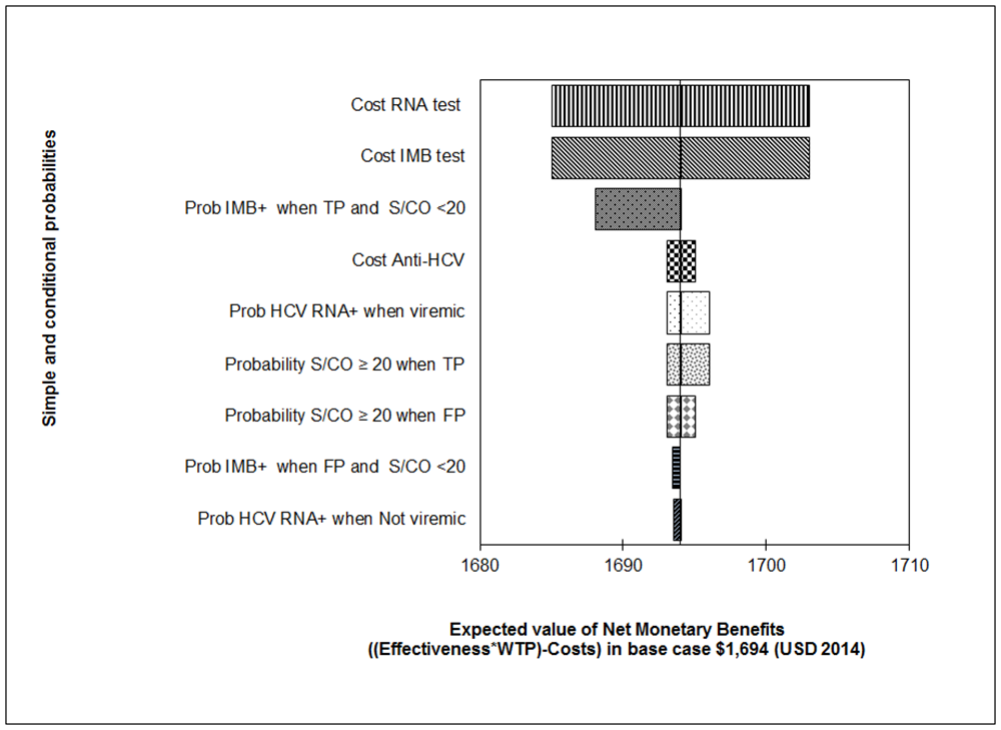
One-way sensitivity analysis tornado diagram. Effects of the parameters with larger influence on the base case estimate of NMB of IMSS-México 3 with an ACER: $ 493 = 185 ÷ 0.375869 considering a WTP of $ 5,000.

Our results from one-way sensitivity analyses suggest that most important parameters for determining the NMB using a WTP of $5,000 USD are: cost of the HCV RNA test, cost of the IMB test, and the probability that the IMB test is positive when the case is a TP and the S/CO<20. Other parameters did not have a significant influence on the base case NMB. (Fig 3).

**Fig 4:**
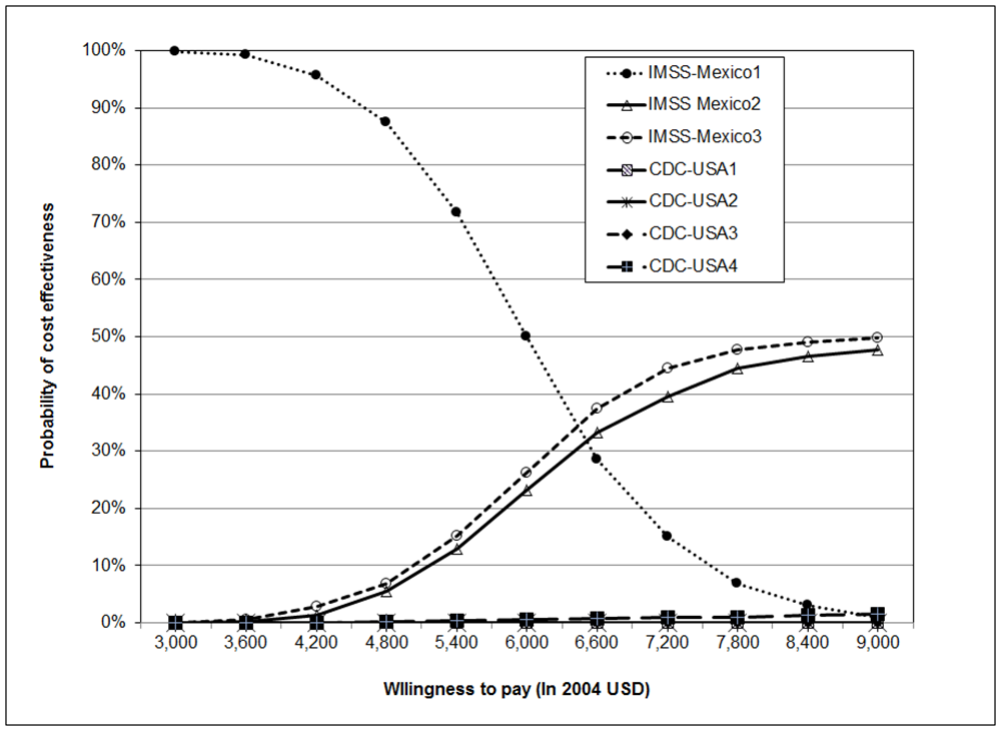
Cost-effectiveness acceptability curves for diagnostic strategies. The CEACs presents the percentage of iterations (resulting from Monte Carlo simulation) accumulated under each strategy for different WTP. All strategies were included in the figure.

Results of PSA suggest that the IMSS-Mexico1 strategy is highly cost-effective when the WTP is below $4,350 USD (Fig 4). Simulation also suggests that the IMSS-Mexico3 and IMSS-Mexico2 strategies are cost-effective and the other four strategies are not cost-effective (Fig 4). We included all alternatives in one graph. For each WTP value, each diagnostic strategy participates in a percentage of the total (100%) of iterations. Another way of graphing CEACs (not presented here) is presenting pairwise alternatives; for example, we could graph IMSS-Mexico3 strategy and IMSS-Mexico1. The result of this comparison is that IMSS-Mexico3 strategy is cost-effective (90% of iterations) at approximately $7,800 USD. The strategy IMSS-Mexico2 is cost effective at $7,900 USD when paired with the IMSS-Mexico1strategy. The CDC-USA3 strategy is cost-effective at a WTP of approximately $8,800 USD. The PSA results suggest that the most cost-effective alternatives considering a WTP of less than $8,800 USD are the IMSS-Mexico3, IMSS-Mexico2, and the CDC-USA3 options.

## Discussion

Our base case scenario findings suggest that the IMSS Mexico3, IMSS Mexico2, and CDC-USA3 strategies have a similar average cost-effectiveness. The PSA results suggest that these three strategies are the most cost-effective for a WTP range between $7,800 and $9,000 USD. For low resource settings, however, the IMSS-Mexico1 option represents an effective and low cost strategy for the diagnosis of chronic HCV infections. The other three strategies we examined (CDC-USA1, CDC-USA2 and CDC-USA4) are less cost-effective, but are more readily available and easier to use because they do not require a three level antibody classification. These options may also be more feasible when the type of immunoassays being used can only report one level, but their incremental cost for detecting an additional case is significantly larger.

The CDC-USA4 strategy is considered the current standard of care for conducting HCV RNA testing in persons who are antibody positive, and is based on the CDC’s new guidelines [6], as well as World Health Organization recommendations [3]. This strategy resulted in an ACER of $651 USD and an ICER of $11,027 USD, and it was dominated by the IMSS-Mexico1 and IMSS-Mexico3 strategies. The annual cost of the IMSS-Mexico1 strategy would be 3.9 million dollars, considering that 30,000 Anti-HCV tests are performed each year at the IMSS blood bank in Guadalajara. The cost of the CDC-USA4 strategy would be 7.26 million dollars. A total of six TP cases would not be detected when using the lower cost IMSS-Mexico1strategy, as compared with the more effective and expensive CDC-USA4 strategy.

Implementation of the IMSS-Mexico1 or any other strategy based on a three-level antibody classification will depend on the availability of the Anti-HCV test reporting data and on the result of the supplemental tests when carrying out the diagnosis. Currently, the two supplemental tests are available at the blood banks in the Mexico City and Guadalajara IMSS medical centers. The HCV RNA test is also used at other non-IMSS specialty hospitals in the public sector, and the IMB test is available in the private sector. However, use of the IMB (RIBA) test is less frequent because it is significantly more expensive, and the current practice at the IMSS blood banks is to use the HCV RNA tests (by nucleic acid testing) when an individual has a positive Anti-HCV test. Compared to IMSS blood banks, the availability of supplemental IMB and HCV RNA testing is more limited in lower resource settings such as second level hospitals, primary care clinics and “Seguro Popular” facilities. Patients can receive these two supplementary tests by being referred to specialty hospitals, through donations from pharmaceutical companies, or by paying out of pocket to private health care providers. Although the IMB test may also be available in some of the aforementioned resource limited settings, the probability of erroneous test results can be greater. The IMB test requires more specialized laboratories and personnel that are properly trained to carry out the testing procedures and interpret the results, than the HCV RNA (NAT) test. IMSS blood banks or specialized laboratories have highly trained personnel and standardized procedures in order to properly execute and interpret the tests.

It is important to note that the various combinations and probabilities of IMB and HCV RNA tests that we used for this study were obtained from an investigation that was conducted at the IMSS blood bank in Guadalajara (9). The present study evaluated the use of seven diagnostic strategies in a sample of healthy blood donors, and it focuses on individuals who have a positive anti-HCV test. The immune system of healthy individuals responds by generating antibodies to HCV during the window period and with viral replication when the infection is expressed. This is not the case in persons with a chronic disease like HIV, or another concomitant chronic disease. In chronically ill individuals, the immune system may not generate a sufficient quantity of antibodies, which can thus result in a negative initial test. However, the infection may be present with HCV viral replication, and thus the probability that the HCV RNA test results are positive is high. In those cases, the antibody level of the anti-HCV test is not useful for diagnosis. Thus, our results are not applicable for these types of populations [7].

The main obstacle for implementing the IMSS-Mexico1 and IMSS-Mexico3 strategies is that the IMB test is not routinely used to identify TP cases. This is a major contradictory issue in the policy of HCV diagnosis because despite its lack of use, the IMB test is still considered one of the main tests that should be used for diagnosis, based on the national official norm of the Ministry of Health [20]. But conversely, the main reason to eliminate the IMB test would be that it is not practical when diagnosing patients who require a prompt test result.

The routine practice in clinical laboratories and blood bank centers is to report a positive HCV antibody test and confirming the results with subsequent HCV RNA testing. Reporting the anti-HCV results as simply reactive or not reactive, as recommended by the CDC, without including the cut-off ratio values may lead to a misunderstanding of the antibody test results. This seems to be an important drawback in the use and interpretation of incomplete test results, and may also lead to inefficiency because the available information is not used appropriately.

This study has some limitations; the first is the lack of data to estimate the probabilities of different immunoassays that may be used in combination with the supplemental tests available in Mexico. The immunoassay we used to estimate the IMSS-Mexico1 and IMSS-Mexico2 strategies is the *ChLIA VITROS* assay. Other types of immunoassays from different providers should be used in future economic analyses.

A second limitation is that the clinical data was collected in 2006, and the technology and use of the three tests has changed since then. However, we believe that the estimated probabilities obtained from more recent data would not be very different from ours because the Guadalajara blood bank study used very high sensitivity and specificity estimates for the IMB and HCV RNA tests. A third limitation is that further validation of the model is necessary. We validated our model through review by clinical experts and discussions with members of our research team. An additional form of validation was the consistency of our results in the sensitivity analyses that were carried out. Further validation of our model is necessary by comparing our results and predictions with data from other similar studies that have collected information from clinical practice. Despite these limitations, our study provides important evidence of more cost-effective ways to confirm a chronic HCV infection in Mexico. Implementing cost-effective testing strategies for HCV is of critical importance in a large, national health care organization, such as IMSS.

## Conclusion

Finding ways to improve the policies and procedures to confirm a chronic infection in patients with a positive Anti-HCV result is critical. Under the current risk-based screening practices, approximately two-thirds of patients will remain undiagnosed until they progress to severe hepatic cirrhosis or liver-related death [3]. Based on the results of our study, our policy recommendations for the diagnosis of chronic HCV infections would be to classify antibodies in two or three levels as measured by the S/CO ratio, and to use the follow-up procedures recommended by the IMSS-Mexico3, IMSS-Mexico2, or CDC-USA3 strategies.

In high-income countries, the main risk factor for HCV is injection drug use. In low-and middle-income countries, the most important risk factors for the nosocomial transmission of HCV are unsafe practices and medical procedures, mainly due to reusing syringes for dispensing intravenous solutions and/or drugs [21, 22]. Although the risk factors for HCV infection are different in low, middle and high-income countries, there is evidence that current diagnosis practices may limit the ability to diagnose chronic HCV patients [23, 24]. Our conclusion is that HCV antibody testing strategies should be based on a classification of two or three antibody levels of the S/CO because they are the most cost-effective way to identify patients who require supplementary testing such as IMB or HCV RNA to confirm a chronic HCV infection.

## Supporting Information

S1 File Estimates of probabilities for CEA model. (XLSX)

S2 File Extended decision tree with probabilities. (TREX)
